# Effect of internal pancreatic duct stent on reducing long-term pancreaticojejunostomy stenosis following pancreaticoduodenectomy

**DOI:** 10.1007/s00423-025-03622-x

**Published:** 2025-01-27

**Authors:** Wei-Hsun Lu, Ying-Jui Chao, Ting-Kai Liao, Ping-Jui Su, Chih-Jung Wang, Yan-Shen Shan

**Affiliations:** 1 Institute of Clinical Medicine, College of Medicine, National Cheng Kung University, 138, Sheng-Li Road, Tainan, 70428 Taiwan; 2 Division of General Surgery, Department of Surgery, National Cheng Kung University Hospital, College of Medicine, National Cheng Kung University, 138, Sheng-Li Road, Tainan, 70428 Taiwan

**Keywords:** Pancreaticoduodenectomy, Pancreaticojejunostomy stenosis, Internal pancreatic duct stent, Pancreatic duct stent migration

## Abstract

**Background:**

As survival following PD improved, long-term complications have emerged as an issue in current era. Pancreaticojejunostomy stenosis is the common long-term sequel after PD but rarely addressed. This study aimed to investigate the benefit of pancreatic duct stent in reducing PJ stenosis after PD.

**Methods:**

Between July 2006 and July 2019, patients undergoing PD with follow-up more than 1 year were recruited. Patients were divided into internal stent, external stent, and no stent groups. We reviewed the Computed tomography (CT) to measure the diameter of pancreatic duct and stent migration at 3 months and 1 year after PD. PJ stenosis was defined as pancreatic duct diameter > 3 mm. Perioperative variables were collected for analysis.

**Results:**

Totally, 506 patients were included 349 patients in internal stent group, 84 patients in the external stent, and 73 patients in no stent group. There was no difference in preoperative P-duct size between the IS and ES group (3.39 ± 1.78 mm vs 3.26 ± 1.89 mm, *p* = 0.481), while the P-duct size was larger in ES group compared to the IS group (3.22 ± 2.44 mm vs. 1.94 ± 2.08 mm, *p* < 0.001) one year after PD. In the internal stent group, the rate of stent migration was 22.1% at 3 months and 67.9% at 1 year post-operatively. CR-POPF (OR 2.24, *p* = 0.015) and P-duct stent retention at PJ > 3 months (OR 0.45, *p* < 0.001) were the independent factors for 1-year PJ stenosis in multivariate analysis.

**Conclusion:**

Retention of internal pancreatic duct stents at the anastomosis for more than 3 months can reduce post-PD PJ stenosis. Extended retention of internal pancreatic duct stents reduces PJ stenosis, highlighting its critical role in preventing long-term complications.

**Supplementary Information:**

The online version contains supplementary material available at 10.1007/s00423-025-03622-x.

## Introduction

Since its first description by Whipple et al. in 1935, pancreaticoduodenectomy (PD) has stood as the primary treatment for periampullary lesions. Initially fraught with a perioperative mortality rate as high as 20% during the 1970s due to its complexity, [1] contemporary high-volume medical centers have significantly reduced surgical mortality to 1% through advancements in surgical techniques and perioperative care [2] The introduction of perioperative chemotherapy has notably extended the lifespan of pancreatic cancer patients undergoing PD [3]. Consequently, long-term complications post-PD have emerged as a new concern for pancreas surgeons in this modern era. Among these complications, pancreaticojejunostomy (PJ) stenosis is critical, leading to chronic pancreatitis and endocrine as well as exocrine insufficiencies [4–6].

Nonetheless, the real incidence of PJ stricture and obstruction after PD would be underestimated since MRCP or ERCP was not routinely performed for postoperative follow-up unless the patients had severe symptoms. Also, the imaging diagnostic criteria for PJ stricture was uncertain. Except for indirect image evidence from MRCP and ERCP, pancreatic duct dilatation not only reflects the patency of PJ but also relates to chronic pancreatitis, exocrine and endocrine insufficiency after PD [7, 8]. Pancreatic duct dilatation was usually defined as the diameter of the main pancreatic duct > 3 mm [5, 7, 9]. Yu et al. also indicated that the patients with exocrine insufficiency were associated with pancreatic duct dilatation(> 3 mm) [6]. Murakami et al. reported that pancreatic duct dilatation caused by PJ stricture or obstruction occurred in 33.3% of PD patients one year after the operation [5] (Supplementary Table 1). Currently, effective strategies to prevent PJ stenosis are lacking. Oida et al. found that duct-to-mucosa anastomosis with pancreatic duct stenting effectively prevents pancreatic leakage and long-term PJ stenosis [10]. Internal and external pancreatic duct stents are widely used in PJ reconstruction, serving as a temporary "transanastomotic bridge," to facilitate pancreatic juice drainage and enhance anastomotic integrity [11]. While previous studies only focus on the short-term outcomes between internal and external P-duct stents [12, 13], the role of PJ stenting in long-term PJ stenosis remains inadequately explored. This article aimed to investigate the long-term outcomes between internal and external stenting in PD, providing more substantial evidence to mitigate long-term complications.

## Methods

### Study design and participants

This single-institutional retrospective study was approved by the Institutional Review Board of National Cheng Kung University Hospital (NCKUH). Between July 2006 and July 2019, a series of 598 consecutive PDs were performed at the NCKUH by the same team led by Professor YS Shan. The data were collected prospectively and analyzed retrospectively. The primary inclusion criterion for selecting subjects was that the patient had survived and received regular follow-up for more than 1 year after PD. The patients enrolled in this study were divided into three groups according to the fashions of transanastomotic pancreatic duct stenting (internal, external, or non-stenting) to clarify the effectiveness of pancreatic duct stenting on the incidence of CR-POPF [14] and long-term pancreatic duct obstruction. Moreover, we recorded the timing of stent removal and migration as well to analyze the association between the duration of the stent retaining and long-term complications. Perioperative clinical data, including age, gender, body mass index (BMI), pancreas gland texture, preoperative pancreatic duct diameter, and pathological diagnosis were collected from the patients’ medical record prospectively.

### Surgical techniques

All patients underwent PJ utilizing the duct-to-mucosa (DTM) technique with 5–0 PDS (polydioxanone) interrupted or continuous sutures. The pancreas capsule was secured to the jejunum serosa using Blumgart techniques [15]. Before 2009, PJ was routinely conducted using external stents. Since 2009, we have predominantly utilized internal stents, reserving external drainage for challenging cases involving extremely small pancreatic duct diameter or soft pancreatic tissue, necessitating complete diversion of pancreatic juice. In the external stent approach, a soft Polyvinyl Chloride (PVC) or silicone suction tube (6 to 12-Fr) was inserted into the main pancreatic duct, with one end protruding through the jejunum to the outside of the abdominal wall. Conversely, in the internal stent method, a PVC or silicone suction tube (6 to 12-Fr) was cut into 6–10 cm lengths and inserted into the main pancreatic duct across the anastomosis. In cases where the pancreatic duct exceeded 5 mm in diameter, DTM without stenting PJ was performed. We routinely checked the amylase level of drainage fluid from peripancreatic space at POD1, 3, 7. The external stent was typically removed between 3 to 9 weeks, depending on the occurrence of PJ leakage.

### Patients follow up

Computed tomography (CT) or magnet resonance imaging MRI would be conducted at the 3rd month, 6th month, first year, and then annually post-PD to confirm the location or absence of the PJ stent and the diameter of pancreatic duct. The diameter of the main pancreatic duct was measured at the widest part of the main pancreatic duct from CT or MRI, and the PJ stenosis was defined as a diameter > 3 mm [5].

### Statistical analysis

Independent t-test was used for comparing the continuous variables with normal distribution between two groups; continuous variables without normal distribution were compared by Wilcoxon rank sum test. Analysis of variance (ANOVA) was used for comparing the continuous variables among three groups and Kruskal–Wallis test was used for comparing three independent samples continuous variables without normal distribution. Categorical variables were analyzed with χ2 test. A logistic regression model was used for analyzing the relationship between a binary outcome and predictor variables. All the statistical analysis was performed with the SAS 9.4 statistical software (SAS Institute Inc. Cary, NC, USA). All test was two-tailed, and *p* < 0.05 was considered as statistically significant. Odds ratios for univariate and multivariate analysis were presented with corresponding 95% confidence intervals.

## Results

**Fig. 1:**
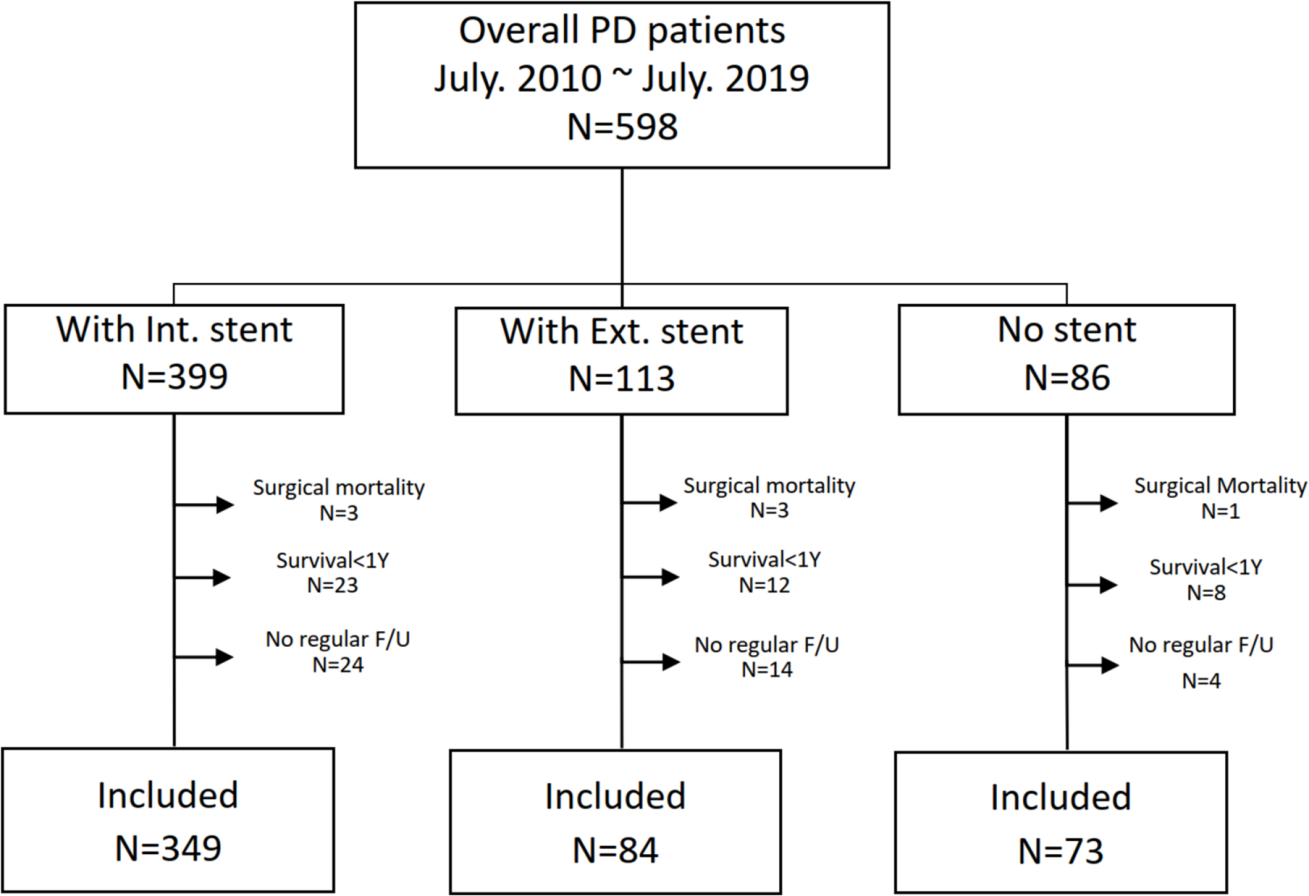
Flow chart of studied patients

Between July 2006 and July 2019, 598 patients underwent PD at National Cheng Kung University Hospital (Supplementary Table 2). Of these, 506 patients who survived and completed more than one year of follow-up were included in this study: 349 with internal PJ stenting, 84 with external PJ stenting, and 73 without PJ stent placement (Fig. 1). The clinicopathologic characteristics of these 506 patients are detailed in Table 1.

**Table 1:** Patient characteristics

Characteristic	Internal stent (*n* = 349)	External stent (*n* = 84)	No stent (*n* = 73)	*P*
Age (mean ± SD), yrs	63.3 ± 11.7	60.3 ± 14.1	61.4 ± 13.0	0.227^a^
Gender (M:F)	205:144	52:32	43:30	0.867^b^
BMI (mean ± SD)	23.8 ± 3.7	23.6 ± 4.0	22.5 ± 3.5	0.022^a^
Pancreas texture				< 0.001^b^
* Soft*	256 (73.4%)	54 (64.3%)	29 (39.7%)	
* Hard*	93 (26.6%)	30 (35.7%)	44 (60.3%)	
Pre-op PD dilatation(> 3mm)	214 (61.3%)	53 (63.1%)	59 (80.8%)	< 0.001^b^
Diagnosis				0.096 ^b^
Malignancy	251 (71.9%)	66 (78.6%)	46 (63.0%)	
* Pancreas head cancer*	119 (34.1%)	28 (33.3%)	29 (39.7%)	
* Distal CBD cancer*	32 (9.2%)	6 (7.1%)	3 (4.1%)	
* Ampulla Vater cancer*	81 (23.2%)	24 (28.6%)	9 (12.3%)	
* Other cancers*	19 (5.5%)	8 (9.5%)	5 (6.8%)	
Benign	98 (28.1%)	18 (21.4%)	27 (37.0%)	
CR-POPF	28 (8.0%)	11 (13.1%)	1 (1.4%)	0.025^b^
*No POPF/Biochemical*	321 (92.0%)	73 (86.9%)	72 (98.6%)	
*grade B*	27 (7.7%)	11 (13.1%)	1 (1.4%)	
*grade C*	1 (0.3%)	0	0	
Hospital stay, median (IQR)	13 (10–18)	25 (20–31)	14 (9–18)	< 0.001^a^
Post-op(> 1yr) PJ stenosis	88 (25.2%)	40 (47.6%)	25 (34.3%)	< 0.001^b^

a, *p*-value was obtained by using Kruskal–Wallis Test; b, *p*-value was obtained by using Chi-square test; *PD* pancreatic duct; *CBD* common bile duct; *CR-POPF* clinically relevant postoperative pancreatic fistula

### Complications of PD among the internal (IS), external (ES), and no stent (NS) group

The incidence of CR-POPF was significantly lower in the NS group (1.4%, *p* = 0.025), yet there was no significant difference between the IS and ES group (8.0% vs 13.1%) (Table 1). The incidence of long-term PJ stenosis was significantly higher in the ES group (47.6%, *p* < 0.001) (Table 1). Among the 153 of 506 patients (30.2%) with postoperative pancreatic duct obstruction, only 5 patients (3.3%) needed further surgical revision of pancreaticojejunostomy. One of these five patients underwent PJ revision due to severe diarrhea and steatorrhea, the other four patients underwent PJ revision due to refractory chronic Pancreatitis.

### The change of the P-duct size and the incidence of the PJ stenosis after PD

**Fig. 2:**
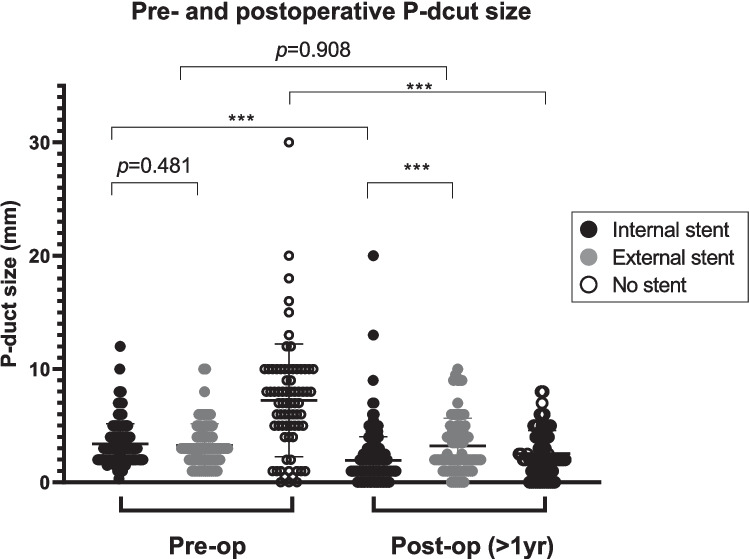
Preoperative and postoperative(> 1 yr) P-duct size. There was no significant difference between the internal and external stent groups (3.39 ± 1.78 vs. 3.26 ± 1.89, *p* = 0.481). Postoperative P-duct sizes were significantly larger in the external stent than internal stent group (3.22 ± 2.44 vs 1.94 ± 2.08. *p* < 0.001). *P* value was obtained by Student’s t test; *** *p* < 0.001

The diameters of the P-duct before PD were largest in the NS group (7.23 ± 4.98 mm, *p* < 0.001), and there was no difference in preoperative P-duct size between the IS and ES group (3.39 ± 1.78 mm vs 3.26 ± 1.89 mm, *p* = 0.481) (Fig. 2).

After a follow-up of over one year, P-duct dilatation significantly resolved in both the IS and NS groups. In the IS group, P-duct diameters decreased from 3.39 ± 1.78 mm to 1.94 ± 2.08 mm (*p* < 0.001), while in the NS group, P-duct diameters decreased from 7.23 ± 4.98 mm to 2.53 ± 2.04 mm (*p* < 0.001). However, there was no difference in P-duct diameters between pre- and post-PD in the ES group (3.26 ± 1.89 mm vs 3.22 ± 2.44 mm, *p* = 0.908). The ES group had significant larger P-duct size when compared to the IS group (3.22 ± 2.44 mm vs. 1.94 ± 2.08 mm, *p* < 0.001) one year after PD (Fig. 2). The ES group has significantly higher incidence of PJ stenosis among the three groups. (*p* < 0.001) (Table 1).

### The duration of P-duct stent retaining and the migration of the internal stent

**Fig. 3:**
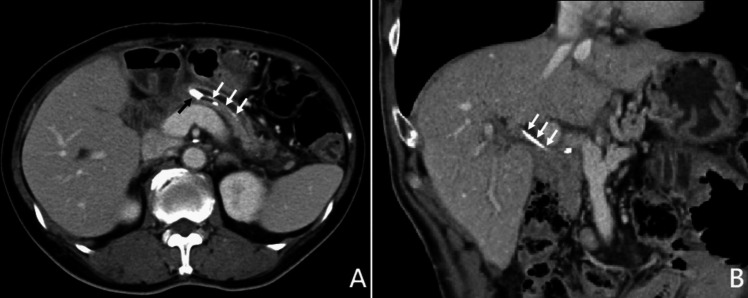
**A** The internal stent (white arrows) migrated into the distal end of the pancreatic duct with P-duct stones formation (black arrow). **B** The internal stent (white arrows) migrated into the common hepatic duct

At 3 months post-PD, 22.1% of patients experienced internal P-duct stent migration, with 10.3% of stents excreted through feces. By one-year post-PD, 43.6% of internal stents were excreted, while 32.1% remained at the PJ anastomoses. At the final follow-up (range: 12 to 62 months, median: 24 months), 62.8% of internal stents had passed out, with only 21.2% remaining at the PJ anastomoses (Table 2). Migration-related complications were rare, including one case (0.3%) of stent migration into the distal pancreatic duct causing P-duct stones and pancreatitis (Fig. 3A), and three cases (0.9%) of stent migration into the common hepatic duct leading to cholangitis (Fig. 3B), managed through endoscopic removal (Table 3).

**Table 2:** Location of internal stent (*n* = 349)

Location	3 M	1Y	End of follow-up
In situ	272 (77.9%)	112 (32.1%)	74 (21.2%)
Passed out of the body	36 (10.3%)	152 (43.6%)	219 (62.8%)
PJ-CJ	32 (9.2%)	51 (14.6%)	23 (6.6%)
CHD	3 (0.9%)	15 (4.3%)	13 (3.7%) ^a^
Distal P-duct	6 (1.7%)	19 (5.4%)	20 (5.7%)

*PJ-CJ* jejunal limb between pancreaticojejunostomy and choledochojejunostomy; *CHD* common hepatic duct; ^a^, 3 patients had the internal stent migrating into the CHD removed via endoscopy

**Table 3:** Relationship between duration of stent stay and PJ stenosis

	≤ 3 M (*n* = 161)	> 3 M (*n* = 272)	*P*
PJ stenosis in 1 year	66 (41.0%)	62(22.8%)	< 0.001^a^
*Pre-op P-duct size (mm)*	3.43 ± 1.96	3.34 ± 1.73	0.068^b^
*Post-op 1year P-duct size (mm)*	3.03 ± 5.41	1.89 ± 2.07	< 0.001^b^

*p*-value was obtained by using ^a^, Chi-square test; ^b^, Student’s t-test; *PJ* pancreaticojejunostomy

### Predictors of long-term PJ stenosis

Analysis of stenosis rates across stent retention duration showed 41.0% for < 3 months (*n* = 161), 22.8% for 3 months to 1 year (*n* = 158), 22.3% for 1–3 years (*n* = 94), 33.3% for 3–5 years (*n* = 15), and 0% for > 5 years (*n* = 5). (Supplementary Table 3) Patients were categorized into two groups based on the duration of PJ stenting: Group 1 (PJ stenting < 3 months) comprised 77 internal and 84 external stenting cases, while Group 2 (PJ stenting > 3 months) included 272 internal stenting cases. Group 2 exhibited a lower incidence of long-term PJ stenosis compared to Group 1 (22.8% vs. 41.0%, *p* < 0.001). Similarly, Group 2 showed smaller pancreatic duct size than Group 1 during long-term follow-up (1.89 ± 2.07 mm vs. 3.03 ± 5.41 mm, *p* < 0.001). Univariate and multivariate analyses revealed CR-POPF as a predictive factor for long-term PJ stenosis (OR 2.24, *p* = 0.015), while P-duct stent retention at anastomoses for over 3 months was protective against long-term PJ stenosis (OR 0.45, *p* < 0.001) (Table 4).

**Table 4:** The association between peri-operative factors and postoperative PJ stenosis

Variables	Univariate		Multivariate	
	Adjusted OR (95% CI)	*p*-value	Adjusted OR (95% CI)	*p*-value
Age	1.004 (0.989–1.020)	0.609		
Male	1.011 (0.688–1.491)	0.955		
Malignant	1.059 (0.697–1.628)	0.790		
Pancreas texture, hard	1.022 (0.680–1.524)	0.917		
CR-POPF	2.241 (1.159- 4.311)	0.015	2.548 (1.256- 5.151)	0.009
Stent in PJ > 3M	0.449 (0.294- 0.684)	< 0.001	0.509 (0.326–0.794)	0.003

Stents in PJ > 3 M, P-duct stents retained at the anastomosis site of pancreaticojejunostomy for more than 3 months; *CR-POPF* clinically relevant postoperative pancreatic fistula; *PJ* pancreaticojejunostomy

## Discussion

In the landscape of pancreaticoduodenectomy (PD) outcomes, the prevention of PJ stenosis remains inadequately addressed in existing literature. Despite the recognized significance of PJ stenosis as a complication, few studies have delved into effective preventative measures. We found that P-duct stent retaining for more than 3 months could decrease the risk of PJ stenosis after PD. To our best knowledge, this study is the largest one that evaluated the risk of long-term PJ stenosis.

There are currently no definitive criteria for diagnosing PJ stenosis, while it typically relies on a combination of clinical manifestation, imaging studies, and sometimes endoscopic or surgical procedures [16]. Pancreatic duct dilatation, typically resulting from PJ stenosis or obstruction after PD, is characterized by main pancreatic duct diameter > 3 mm, indicating PJ stenosis [5, 7]. Murakami et al. reported pancreatic duct dilatation caused by PJ stenosis in 33.3% of PD patients one-year postoperatively [5]. However, the reported rates of symptomatic PJ stenosis diagnosed by MRCP or ERCP range from 2 to 11% [8, 17]. This variability may be due to the infrequent use of MRCP or ERCP in follow-up for asymptomatic patients, and uncertain imaging diagnostic criteria, potentially leading to an underestimation of PJ stenosis incidence post-PD. In our study, 155 of 506 (30.6%) patients experienced postoperative PJ stenosis, comparable with previous report [5].

Unlike the POPF, which would lead to immediate and severe clinical consequences, the complications related to postoperative PJ stenosis usually did not have acute significant manifestation. However, PJ stenosis may cause not only postoperative chronic pancreatitis and pancreatic duct calculi [18], but also exocrine and endocrine insufficiency and further pancreatic remnant atrophy [5, 19–21]. Vanbrugghe et al. reported that 45% of patients with PJ stenosis were asymptomatic, while 36% exhibited endocrine insufficiency, 27% showed exocrine insufficiency, and 45% presented radiological signs of pancreatitis [16]. Exocrine insufficiency tends to be more prevalent than endocrine insufficiency in these cases. The overall exocrine function of the pancreatic remnant depends on the amount and the functional reserves of residual parenchyma, and on the pancreatic duct outflow [21]. The obstruction of the pancreatic duct and impair the drainage of pancreatic juice into the jejunum. This obstruction can lead to inflammation, fibrosis, and ultimately atrophy of the pancreatic parenchyma. Exocrine insufficiency commonly manifests as diarrhea. Nordback er al. reported that at least half of post-PD diarrhea were associated with PJ stenosis [21]. Moreover, Yu et al. reported that post-PD exocrine insufficiency can hinder the release of zinc from food, and serum zinc level was significantly negatively correlated with pancreatic duct diameter [6]. However, no evidence suggested that pancreatic exocrine and endocrine insufficiency can be improved through any intervention. Surgical intervention should be reserved for cases of refractory pancreatitis caused by PJ stenosis [22].

The pathophysiology of long-term PJ stenosis is not fully understood. Ischemia, a known risk factor for stenosis in other gastrointestinal tract anastomoses, may also contribute to PJ stenosis [23, 24]. Surgeons often employ techniques like suture ligation or electrocoagulation during PJ reconstruction to prevent bleeding, potentially leading to ischemia at the anastomotic site. Strasberg et al. found insufficient blood supply to the remnant pancreas in 38% of PD patients during reconstruction [25]. Aside from ischemia, other risk factors for long-term PJ stenosis post-PD remain unidentified [8]. Exclusion of the no stent group due to the specific nature of dilated pancreatic duct, we found that CR-POPF was associated with PJ stenosis (OR = 2.548, *p* = 0.009). This may be because pancreatic fistula can lead to inflammation, infection, and scarring around the PJ site, potentially increasing the risk of long-term PJ stenosis. As we know, this risk factor has not been previously reported.

Furthermore, in our study, the incidence of PJ stenosis in the external stent group was higher than in the internal stent group (47.6% vs. 25.2%). This protective effect was existed only when the internal stent retains in the PJ for more than 3 months. A plausible explanation is the scar formation mechanism. Hypertrophic scars typically develop over 3 to 6 months, and retained stent can prevent scar contracture in the period [26, 27]. Previous studies on biliary strictures indicate that prolonged stenting promotes scar remodeling and enhances anastomosis patency [28, 29]. This may explain why the internal stents retained in the PJ for more than three months can reduce the risk of PJ stenosis.

While internal stents exhibit a lower incidence of PJ stenosis, they still pose other potential risks, including stent migration. In our study, one patient (0.3%) had the internal stent migrating into the distal part of the pancreatic duct leading to P-duct stones and pancreatitis, and 3 patients (0.9%) with the internal stent migrating into the common hepatic duct (CHD) inducing cholangitis and removed through endoscopic approach. The future of P-duct stent design is set to prioritize both incorporating biodegradable materials and preventing early migration. Bakheet et al. introduced a biodegradable tubular stent (BTS) utilizing a terpolymer blend. Their rat studies demonstrated complete BTS degradation within 12 weeks, while in a porcine PJ model, the BTS group exhibited significantly smaller luminal diameter and pancreatic duct area compared to controls [30]. Also, incorporating anchor mechanisms may help prevent early migration. By combining these features, an optimal P-duct stent design should be engineered to remain within the pancreatic duct for over 3 months, offering support during the crucial healing phase following PD.

This study has several limitations. It is a single-center retrospective investigation; however, it distinguishes itself with a considerable case volume. The retrospective design introduces patient selection bias, with surgeons more likely to opt for external stenting in cases of challenging PJ anastomoses to mitigate CR-POPF risk [13]. This selection bias may be associated with a higher incidence of short-term and long-term complications in the external stent group. Additionally, our study lacks data on endocrine and exocrine function, highlighting the need for further functional investigations to demonstrate the clinical significance of long-term PJ stenosis."

In conclusion, we identified that CR-POPF and early P-duct stent migration or removal within 3 months are the major risk factors for long-term pancreatic duct obstruction. Our findings can serve as a foundation for further refinement in the design of P-duct stents.

## Supplementary Information

Below is the link to the electronic supplementary material.

Supplementary file1 (DOCX 24 KB)

## Data Availability

No datasets were generated or analysed during the current study.
