# Supplementary material for: Effect of internal pancreatic duct stent on reducing long-term pancreaticojejunostomy stenosis following pancreaticoduodenectomy
Source: Langenbecks Arch Surg. 2025 Jan 27;410(1):51. doi: 10.1007/s00423-025-03622-x (PMC11772507; doi:10.1007/s00423-025-03622-x)
Supplement: Supplementary file 1 — Supplementary file1 (DOCX 24 KB) [file 423_2025_3622_MOESM1_ESM.docx]

| **Supplementary table1.** Summary of published literature about the clinical significance of P-duct dilatation and PJ stenosis | | |
| --- | --- | --- |
| Author/Journal | Parameters for PJ stricture (Indirect image evidence) | Impact of functional status associated with PJ complications |
| Murakami *et al.* [5]  Surg Today (2017) | P-duct dilatation**:**  Diameter of main P-duct >3 mm | 44 of 132 (33.3%) PD patients developed P-duct dilatation.  P-duct dilatation associated with a higher atrophy rate (34.9%), lower level of serum Albumin and body weight gain |
| Zhang *et al.* [7]  BMC Gastroenterology (2018) | P-duct obstruction**:**  Diameter of main P-duct >3 mm | 25 of 78 (32.1%) patients had post-PD P-duct obstruction  P-duct obstruction associated with higher rate of exocrine and endocrine insufficiency, and pancreatitis (52% vs. 9.4%, p<0.001) |
| Yu *et al.* [6]  World J Surg (2011) | Pancreatic duct-parenchymal ratio (DPR)^a^ **:**  Main P-duct diameter (>3mm) | 33 of 48 (68%) patients had a zinc deﬁciency (Serum zinc levels<80 mcg/dl)  Zinc-deﬁcient group (*vs. Zn-sufficient group*) had  - Higher DPR (30.1% vs. 21.2%, p=0.005)  - Dilated P-duct diameter (4.36mm vs. 3.20mm, p=0.028)  -Higher rate of stool fat positive (26.3% vs. 0%, p=0.038) |
| Demirjian *et al.* [8]  HPB (Oxford) (2010) | Symptomatic PJS  -Filling defect at the PJ under secretin-enhanced MRCP  -Abdominal pain | 7 of 357 (2%) PD patients developed symptomatic PJS. |
| Morgan *et al.* [17]  HPB (2010) | PJ stenosis  -Filling defect at the PJ under secretin-enhanced MRCP  - Abdominal pain | 27 of 237 (11.4%) PD patients for benign disease developed PJ stenosis and needed revision |
| a, Pancreatic duct-parenchymal ratio (DPR) are measured at the ﬁrst lumbar spine (L1) level on an abdominal CT or MRI scan | | |

| **Supplementary table 2.** Overall patient characteristics and operative results | | | |
| --- | --- | --- | --- |
| Characteristics | | n=598 | |
| Age (mean±SD), yrs | | 63.1±12.6 | |
| Gender (M:F) | | 353:245 | |
| BMI (mean±SD) | | 23.5±3.6 | |
| Operation method | |  | |
| *Whipple operation* | | 218 (36.5%) | |
| *PPPD* | | 380 (63.5%) | |
| ASA | |  | |
| *I* | | 17 (2.8%) | |
| *II* | | 278 (46.5%) | |
| *III* | | 290 (48.5%) | |
| *IV* | | 13(2.2%) | |
| Tumor size (cm) | | 3.0±1.8 | |
| Pancreas texture | |  | |
| *Soft* | | 397 (66.4%) | |
| *Hard* | | 201 (33.6%) | |
| OP time (mean±SD), min | | 287±105 | |
| Blood loss (mean±SD), ml | | 380±467 | |
| Diagnosis | |  | |
| Malignancy | | 426 (71.2%) | |
| *Pancreas head cancer* | | 206 (34.4%) | |
| *Distal CBD cancer* | | 48 (8%) | |
| *Ampulla Vater cancer* | | 131 (21.9%) | |
| *Duodenal cancer* | | 13 (2.2%) | |
| *Other cancers* | | 28 (4.7%) | |
| Benign | | 172 (28.8%) | |
| *IPMN* | | 39 (6.5%) | |
| *Neuroendocrine tumor* | | 11 (1.8%) | |
| *Chronic pancreatitis* | | 47 (7.9%) | |
| *Other benign lesions* | | 75 (12.5%) | |
| Complication (Clavein-Dindo) | |  | |
| *0* | | 306 (51.2%) | |
| *Grade I* | | 38 (6.4%) | |
| *Grade II* | | 154 (25.8%) | |
| *Grade IIIa* | | 44 (7.4%) | |
| *Grade IIIb* | | 17 (2.8%) | |
| *Grade IVa* | | 16 (2.7%) | |
| *Grade IVb* | | 16 (2.7%) | |
| *Grade V* | | 7 (1.2%) | |
| **CR-POPF** | | **53 (8.9%)** | |
| ***grade B*** | | **47 (7.9%)** | |
| ***grade C*** | | **6 (1.0%)** | |
| PPH (grade B/C) | | 61 (10.2%) | |
| DGE (grade B/C) | | 108 (18.1%) | |
| Bile leakage | | 67 (11.2%) | |
| Fluid accumulation | | 92 (15.4%) | |
| Wound infection | | 90 (15.1%) | |
| Hospital stay, median (IQR) | | 16 (12-24) | |
| SD, standard deviation; M, male; F, female; BMI, body mass index; PPPD, pylorus-preserving pancreaticoduodenectomy; ASA, The American Society of Anesthesiologists Physical Status Classification ; CBD, common bile duct; IPMN, Intraductal papillary mucinous neoplasm; CR-POPF, clinically relevant postoperative pancreatic fistula; PPH, post-pancreatectomy hemorrhage; DGE, Delayed gastric emptying | | | |

| **Supplementary table 3.** Relationship between duration of stent stay and PJ stenosis | |
| --- | --- |
| Duration of Stent stay | Long-term PJ stenosis rate |
| *<3 months* | *41.0% (66/161)* |
| *3 months - 1 year* | *22.8% (36/158)* |
| *1-3 years* | *22.3% (21/94)* |
| *3-5 years* | *33.3% (5/15)* |
| *>5 years* | *0% (0/5)* |
